# Pregnancy-Induced Hypertension is Accompanied by Decreased Number of Circulating Endothelial Cells and Circulating Endothelial Progenitor Cells

**DOI:** 10.1007/s00005-014-0278-x

**Published:** 2014-02-23

**Authors:** Jerzy Heimrath, Maria Paprocka, Andrzej Czekanski, Agata Ledwozyw, Aneta Kantor, Danuta Dus

**Affiliations:** 1 Department of Gynaecology and Obstetrics, Faculty of Health Science, Wrocław Medical University, Wrocław, Poland; 2 Ludwik Hirszfeld Institute of Immunology and Experimental Therapy, Polish Academy of Sciences, Rudolfa Weigla 12, 53-114 Wrocław, Poland; 3 Second Department and Clinic of Gynaecology, Obstetrics and Neonatology, Wrocław Medical University, Wrocław, Poland

**Keywords:** CECs, EPCs, Preeclampsia

## Abstract

Maternal endothelial dysfunction is one of the main features of pregnancy-induced hypertension (PIH). It is generally accepted that circulating endothelial cells (CECs) and endothelial progenitor cells (EPCs) reflect the state of the endothelium, its injury and/or repair possibilities. The objective of this study was to determine whether the CECs and EPCs numbers in the circulation of women with PIH reflect the presence of this pathology. Peripheral blood cells of PIH and normotensive pregnant women were labeled with specific monoclonal antibodies. For CECs evaluation, samples were labeled with anti-CD31 and anti-CD45 antibodies; for EPCs with anti-VEGFR2/KDR and anti-CD34 antibodies. Cells were quantified by flow cytometry. The levels of both CECs (CD31^+^, CD45^−^) and EPCs (CD34^+^, VEGFR2/KDR^+^) in the peripheral blood of women with PIH were significantly lower compared with those of control pregnant women with normal blood pressure level. Lowered accessibility of maternal CECs and EPCs may diminish general regenerative potential of the patient endothelia, contributing to PIH symptoms and to the risk of subsequent coronary and arterial disease.

## Introduction

According to current opinion, arterial pregnancy-induced hypertension (PIH) is caused by insufficient placental flow. In PIH trofoblast fails to modulate its phenotype, which results in impaired, shallow invasion of spiral arteries. Reduced uteroplacental perfusion induces series of hypoxia/ischemia events, accompanied with the imbalance between vasodilative and the vasopressive factors, a dysfunction of the renin–angiotensin system as well as endothelium activation. This activation is not restricted to placental vessels but affects also other maternal endothelia, making PIH a multisystemic disease. Preeclampsia is a pregnancy pathology in which PIH is accompanied by proteinuria and associated with an increased risk of subsequent coronary and arterial diseases (Blum et al. 2003; Gilbert et al. 2008; Harskamp and Zeeman 2007; Kaufmann et al. 2003; Robb et al. 2007; Zhang et al. 1997).

Dysfunction of vascular endothelium in PIH women is accompanied by elevated amounts of syncytiotrophoblast particles and increased levels of several soluble adhesion molecules, e.g., sVCAM and sICAM, in peripheral blood (Heimrath et al. 2004). Other studies have shown that concentration of serum vascular endothelial growth factor (VEGF), von Willebrand factor and fibronectin was also elevated. Levels of soluble markers, tumor necrosis factor, endothelin and angiotensin II, may also be elevated whereas the vasodilator levels, nitric oxide and prostacyclin levels, are usually diminished (Blum et al. 2003; Gammill et al. 2007; Gilbert et al. 2008; Hunter et al. 2000; Kwon et al. 2007).

Circulating endothelial cells (CECs) and endothelial progenitor cells (EPCs) reflect the state of the endothelium, its injury and/or repair possibilities (Hunting et al. 2005). CECs are cells peeled off from the vessel wall during normal or pathological conditions, such as acute inflammation or mechanical trauma. CECs are usually defined as cells expressing endothelial cell markers, such as CD146, CD31 or Willebrand factor, but negative for CD45 (Blann et al. 2005; Khan et al. 2005; Mancuso et al. 2001; Monestiroli et al. 2001; Woywodt et al. 2002).

Several studies have demonstrated that EPC may participate in the postnatal neoangiogenesis. These cells are characterized by expression of such markers as CD133, CD34 and VEGFR2 (VEGF receptor 2, also known as KDR) (Hristov and Weber 2004; Hunting et al. 2005; Robb et al. 2007; Timmermans et al. 2009).

The aim of this study was to determine whether the CECs and EPCs numbers in the PIH patients may reflect the presence of this pathology.

## Materials and Methods

### Subjects

The study involved 18 pregnant women, 21–35 years, with PIH, defined according the criteria of blood pressure elevation >140/90 mmHg, measured at least twice, 6 h apart, after 20th week of gestation in a previously normotensive women. Dopegyt (Methyldopa) and Dihydralzine were applied as a standard antihypertension treatment. Preeclampsia was diagnosed in six women (30 %) with PIH, on the basis of the presence of 300 mg of protein or more in a 24-h urine sample. In the group studied, ten women (55.5 %) were primigravida and eight women (44.5 %) were multipara. Seven of them (38.8 %) had premature labor (before the end of the 37th week of pregnancy). The control consisted of 21 pregnant women aged 22–43 years, with normal blood pressure level, having natural labor in term; and included 10 (47.5 %) primigravida and 11 (52.5 %) multipara. All women had monovular pregnancies. Main clinical characteristics of both patient groups were gathered in a Table 1. Pregnant women in both groups were carefully selected. There was no patient with HELLP syndrome (preeclampsia variant, characterized by hemolysis, elevated liver enzymes and low platelet count). Patients with overweight, cigarette smoking, diabetes or hypertension were not included in the group. The main difference between control and PIH groups was average delivery time: 40 vs. 37 weeks, respectively, as in PIH pregnancy usually there is a need of a delivery before the term because of complications related to hypertension.

**Table 1:** Characteristics of study groups

	Control	PIH patients
Number of patients	21	18
Age	28 (±5)	30 (±9)
Blood pressure (mm Hg)		
Systolic	122 (±10)	174 (±15)
Diastolic	79 (±12)	110 (±15)
Proteinuria	0/21	6/18
Gestational age at the blood sampling (week)	40 (±2)	37 (±4)
Natural birth	21/21	2/18
Cesarean section	0/21	16/18
Infantile birth weight (g)	3,433.6 (±400)	2,934.6 (±500)
Rate of primipara	47.5 %	55.5 %

The material examined was venous blood drawn directly before birth into test tubes with an anticoagulant. The Bioethics Commission of the Wroclaw Medical University approved this study and all patients gave informed consent.

### Flow Cytometry Analysis of CECs and EPCs Numbers in Peripheral Blood

To determine CECs number, blood samples of PIH patients and normotensive pregnant women were labeled with FITC-conjugated anti-CD31 and PerCP-conjugated anti-CD45 antibodies (BD Biosciences, USA). To evaluate EPCs number PerCP-conjugated anti-CD34 (BD Biosciences, USA) and PE-conjugated anti-VEGFR2/KDR antibodies (R&D Systems, USA) were used. Blood samples were incubated first with FcR-blocking reagent (Miltenyi Biotec, Germany) and then with selected antibodies. Isotype matched, labeled immunoglobulins (BD Biosciences, USA or R&D Systems, USA, respectively) were used as a control. After incubation Lysing Solution (Sigma, Germany) was applied to eliminate erythrocytes and fluorescent CytoCount beads (Dako, Denmark) were used for cell number evaluation. Minimal numbers of 150,000 cells were collected. Flow cytometry was performed using FACSCalibur cytometer, CellQuest software (Becton–Dickinson, USA) and appropriate cell gating to eliminate platelets and dead cells (debris gate). EPCs were evaluated from lymphocyte gate (Redondo et al. 2008) and CECs from mononuclear cells gate (Mancuso et al. 2001; Monestiroli et al. 2001; Redondo et al. 2008). Data were expressed as a cell count per 1 mL of blood. Cell numbers obtained by flow analysis for control normotensive group and PIH patients were compared using Mann–Whitney *U* test (Statistica 6.0 software). Differences were considered as statistically significant when *p* < 0.05.

## Results and Discussion

**Fig. 1:**
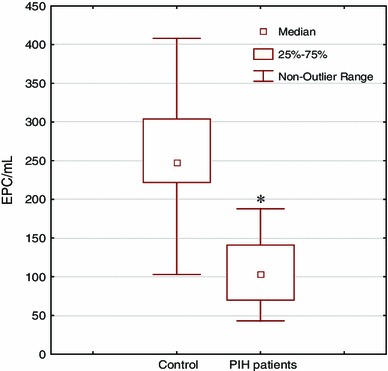
The numbers of endothelial progenitor cells (EPCs) in 1 mL of blood of normotensive pregnant woman and patients with pregnancy-induced hypertension (PIH) evaluated by flow cytometry. Significantly decreased cell numbers were observed in PIH patients: 1.03 × 10^2^ when compared with normal pregnant control: 2.48 × 10^2^ per 1 mL (*p = 0.000001)

**Fig. 2:**
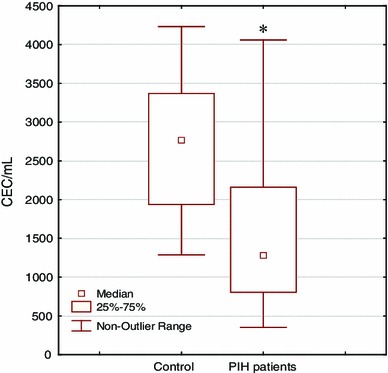
The numbers of circulating endothelial cells (CECs) in 1 mL of blood of normotensive pregnant woman and patients with pregnancy-induced hypertension (PIH) evaluated by flow cytometry. Significantly decreased cell numbers were observed in PIH patients: 1.28 × 10^3^ when compared with normal pregnant control: 2.77 × 10^3^ per 1 mL (*p = 0.0034)

As the endothelium dysfunction plays a significant causal role in PIH, we have decided for evaluation, in the same patients, both EPCs and CECs to receive more complete insight into the problem. Pregnant women with PIH were found to have approximately twice lower counts of both EPCs and CECs as compared to normotensive healthy controls. An average EPCs number in peripheral blood of healthy controls was 2.48 × 10^2^ but in PIH patients it decreased to 1.03 × 10^2^ per 1 mL (*p* = 0.000001; Fig. 1). CECs counts were 2.77 × 10^3^ in healthy controls and 1.28 × 10^3^ per 1 mL in PIH patients (*p* = 0.0034; Fig. 2). Obtained differences were statistically significant.

The process of neoangiogenesis means in situ proliferation of local endothelial cells but involves also EPCs mobilized from bone marrow (Hristov and Weber 2004). To pass over a lack of specific marker for EPC, two, three or even more markers have to be applied to discriminate these from platelets and lymphocytes (Robb et al. 2007; Timmermans et al. 2009).

Our data confirm a few published data that EPCs level in PIH women was diminished. Sugawara et al. (2005) reported decrease in EPCs number and found EPCs cellular senescence. Kwon et al. (2007) also found decreased number of EPCs in preeclampsia. Gammill et al. (2007) have reported that EPCs number increases in healthy pregnancy but similar increase was not observed in woman with preeclampsia. Only Matsubara et al. (2006) did not find significant differences in EPCs levels.

CECs number in healthy people, reported in the literature, ranges from 0 to 7,900 per 1 mL of blood (Khan et al. 2005; Steurer et al. 2008). Our data concerning CECs differ from those from other laboratories. We have found lower counts of CECs in PIH patients, compared to normotensive controls, while publications by Zheng et al. (1996) and Grundmann et al. (2008) reported augmented number of CECs in PIH patients. However, their CECs enumerating methods were different. Zeng’s group isolated CECs before their counting under microscope, and Grundmann’s group separated CECs with anti-CD146 antibody and stained cells with Ulex lectin, which may result in enumeration of different cell subpopulations. All ten patients evaluated by Grundman’s study were patients with preeclampsia. PIH and preeclampsia patients were presented together in our study, as we had not found any difference in CECs number in these groups.

It seems also possible that some of CECs released from activated endothelium disintegrate and form microparticles (EMs), small vesicles of endothelial cell membrane, containing cytoplasm. In preeclampsia EMs levels, evaluated by flow cytometry as CD62^+^ and CD31^+^ CD45^−^ microparticles, were significantly higher than in healthy controls (Gonzalez-Quintero et al. 2004). In our study microparticles were not specifically labeled nor evaluated but by gating living cells for CEC and EPC counting, we could observe augmented debris number in PIH patients (data not shown). This suggests excessive cell’s damage which may influence final CECs count.

Because EPCs and CECs levels in the peripheral blood of women with PIH were found to be significantly lower as compared with those of normotensive pregnant women we suggest that this phenomenon often accompanied the pathology. Patients selected for our study were without overweight, diabetes or hypertension but decreased number of EPC and CEC numbers may be associated with other pathological processes such as insulin resistance, which was also demonstrated to impair functions of these cells (Thadhani et al. 2004).

One may assume that lower accessibility of these maternal cells impairs regenerative capabilities of the endothelium and may be one of the factors contributing to the development of pregnancy-induced hypertension. In addition, general endothelial dysfunction and lowered number of cells able to restore vascular bed may give, as a remote effect, augmented risk of cardiovascular diseases, as it was demonstrated recently (Harskamp and Zeeman 2007).

## Abbreviations

CECs: Circulating endothelial cells
EPCs: Endothelial progenitor cells
PIH: Pregnancy-induced hypertension
